# FGF4, A New Potential Regulator in Gestational Diabetes Mellitus

**DOI:** 10.3389/fphar.2022.827617

**Published:** 2022-03-04

**Authors:** Miaojuan Fan, Tongtong Pan, Wei Jin, Jian Sun, Shujun Zhang, Yali Du, Xinwei Chen, Qiong Chen, Wenxin Xu, Siew Woh Choo, Guanghui Zhu, Yongping Chen, Jie Zhou

**Affiliations:** ^1^ Department of Infectious Diseases & Zhejiang Provincial Key laboratory of Liver Diseases, The First Affiliated Hospital of Wenzhou Medical University, Wenzhou, China; ^2^ School of Pharmaceutical Sciences, Wenzhou Medical University, Wenzhou, China; ^3^ Baoji Maternal and Child Health Hospital, Baoji, China; ^4^ The Fourth Affiliated Hospital, Zhejiang University School of Medicine, Jinhua, China; ^5^ The Second Affiliated Hospital and Yuying Children’s Hospital of Wenzhou Medical University, Wenzhou, China; ^6^ College of Science and Technology, Wenzhou-Kean University, Wenzhou, China

**Keywords:** gestational diabetes mellitus, FGF4, embryopathy, neural tube defects, placenta

## Abstract

**Background:** Gestational diabetes mellitus (GDM) is associated with adverse maternal and neonatal outcomes, however the underlying mechanisms remain elusive. The aim of this study was to find efficient regulator of FGFs in response to the pathogenesis of GDM and explore the role of the FGFs in GDM.

**Methods:** We performed a systematic screening of placental FGFs in GDM patients and further in two different GDM mouse models to investigate their expression changes. Significant changed FGF4 was selected, engineered, purified, and used to treat GDM mice in order to examine whether it can regulate the adverse metabolic phenotypes of the diabetic mice and protect their fetus.

**Results:** We found FGF4 expression was elevated in GDM patients and its level was positively correlated to blood glucose, indicating a physiological relevance of FGF4 with respect to the development of GDM. Recombinant FGF4 (rFGF4) treatment could effectively normalize the adverse metabolic phenotypes in high fat diet induced GDM mice but not in STZ induced GDM mice. However, rFGF4 was highly effective in reduce of neural tube defects (NTDs) of embryos in both the two GDM models. Mechanistically, rFGF4 treatment inhibits pro-inflammatory signaling cascades and neuroepithelial cell apoptosis of both GDM models, which was independent of glucose regulation.

**Conclusions/interpretation:** Our study provides novel insight into the important roles of placental FGF4 and suggests that it may serve as a promising diagnostic factor and therapeutic target for GDM.

## Introduction

Gestational diabetes mellitus (GDM) is a condition of glucose intolerance which is first recognized during pregnancy, affecting up to 15% of pregnancies worldwide (American Diabetes Association, 2014; Yeral et al., 2014). It is associated with adverse maternal and neonatal outcomes. GDM exacerbates excessive fetal growth, dystocia, and malformation (Hapo Study Cooperative Research Group et al., 2008). Hypoxia, hyperglycemia, and hyperinsulinemia are risk factors for GDM and may adversely affect placentation and development of the maternal-fetal vascular exchange (Liao et al., 2016). The placenta serves as a springboard between the mother and fetus, playing a pivotal role in mediating maternal physiological changes and fetal development (Enquobahrie et al., 2009). It produces many hormones and growth factors with endocrine and paracrine effects, secreting into both maternal and fetal blood, depending on the properties of the hormone. Interestingly, it has be reported that insulin resistance during pregnancy may be attributed to the release of placental hormones (Barbour et al., 2007). Several lines of evidence point to a pivotal role of placental hormones and inflammatory cytokines in the pathological process of GDM (Kirwan et al., 2002; Menon and Taylor, 2019). In addition, as a serine/threonine kinase that is conserved within eukaryotes, AMPK, is necessary for the proper placental differentiation, nutrient transportation, maternal and fetal energy homeostasis, and the protection of the fetal membrane in the process of pregnancy (Carey et al., 2014).

The fibroblast growth factor (FGF) family consists of 18 polypeptides that are critically involved in a myriad of biological functions such as cell growth and differentiation, angiogenesis, embryonic development, wound healing and repair, as well as metabolic regulation (Turner and Grose, 2001). Although they are structurally related, FGFs has been grouped into six homologous subfamilies in a paracrine or endocrine manner (Beenken and Mohammadi, 2009; Goetz and Mohammadi, 2013). It has been reported that two endocrine FGFs (i.e. FGF21 and FGF19) can potentially regulate metabolic disorder in gestational diabetes (Dongyu et al., 2013; Dongyu and Wenjing, 2019). The FGF21 expression in human placenta is protectively elevated in GDM and affects placental metabolism and nutrient transfer and thereby the fetal growth. It can be secreted directly into the fetal circulation and affect fetal metabolism (Dekker Nitert et al., 2014a). With the discovery of the metabolic function of paracrine FGF1 (Jonker et al., 2012; Suh et al., 2014; Gasser et al., 2017; Lin et al., 2021), other paracrine FGFs may also have metabolic potential. However, their roles in GDM have not been studied. Therefore, it is important to thoroughly examine whether paracrine FGFs can function as regulators in the development of GDM.

In this study, we comprehensively analyzed six representative FGFs from six FGF subfamilies in both normoglycemic pregnancies and the GDM placenta-affected pregnancies. We found that FGF4 expression was elevated in the placentas from GDM patients and its level was correlated to glucose, indicating its important physiological or pathological role in GDM. To test the hypothesis that it may play a protective role to adapt blood glucose stress, like FGF21, we constructed two typical mouse models of diabetes during pregnancy, to mimic human GDM. Our study showed that human recombinant FGF4 (rFGF4) improved maternal glucose tolerance in high fat diet (HFD) induced gestational diabetes mice, and decreased neural tube defects (NTDs) of embryos in both HFD induced and streptozotocin (STZ) induced gestational diabetes mice. Furthermore, rFGF4 exert this effect by inhibiting placental inflammation with consequent reduction of neuroepithelial cell apoptosis, which was independent of glucose regulation.

## Materials and Methods

### Study Population and Samples

A cohort was established from pregnant women attending their first routine visit at an obstetric outpatient department between November 2017 and September 2019. Pregnant women who were at 24–28 weeks of gestation underwent blood glucose screening according to the clinical criteria of GDM after providing written informed consent. Women with fasting plasma glucose level ≥5.1 mmol/L or ≥8.5 mmol/L 2 h after an oral glucose load (75 g) were diagnosed with GDM (Homko, 2011; World Health Organization, 2013). They were non-smokers and had singleton, normotensive pregnancies and without intrauterine infection or any other medical or obstetric complications except GDM. The central areas of the placentas were dissected immediately after surgeries from all patients according to the approved protocols by the ethics committees of First Affiliated Hospital of Wenzhou Medical University.

### Mice

All female and male C57BL/6J mice were purchased from the Model Animal Research Center of Nanjing University (Nanjing, China). They were housed in an animal facility with a controlled environment (22 ± 2 °C, 50%–60% humidity, 12-h light/dark cycle, and lights on at 0700 h) and free access to food and water. All procedures for handling animals were approved by the Institutional Animal Care and Use Committee of Wenzhou Medical University and conformed to the Guide for the care and use of laboratory animals published by the National Institutes of Health (NIH; publication No. 8023, revised 1978).

### HFD Induced GDM Model

Four-week-old C57BL/6J female mice were divided into two groups and fed either a HFD (60% fat; Research Diets, Inc., Harlan, United States) or a normal diet (10% fat; Research Diets, Inc., Harlan, United States) for 10 weeks. Following the 10-weeks feeding regimen, blood glucose levels were determined using FreeStyle complete blood glucose monitor (Abbott Diabetes Care Inc., Alameda, CA). Mice from the HFD group continuously gained weight starting from weeks 2–10. They were considered as HFD induced GDM model when the mice blood glucose ≥240 mg/dl (13.3 mM).

Female mice were then mated with C57BL/6J male mice at 3:00 PM. Embryonic day 0.5 (E is the abbreviation of embryonic day) was designated once the vaginal plug was present the next morning. Long-acting semaglutide (Novo Nordisk, Novo Alle, Denmark) were subcutaneously injected once in diabetic mice prior to mating to restore euglycemia to protect early embryonic formation and implantation. The developing embryos were exposed to a hyperglycemic condition from E5.5 to E10.5, which is a critical period for early neural tube development. The non-diabetic female C57BL/6J mice (10% fat; research diets, Harlan, United States) were injected with semaglutide once before mating served as non-diabetic controls.

### STZ Induced GDM Model

All wild-type (WT) female C57BL/6J mice aged 7-week old were intraperitoneally injected daily with 60 mg/kg STZ (Sigma-Aldrich, Saint Louis, Missouri, United States) over 5 days to induce diabetes. The STZ-injected mice were considered as having diabetes when their blood glucose were greater than or equal to 300 mg/dl (16.7 mM). Long-acting semaglutide (Novo Nordisk, Novo Alle, Denmark) was then subcutaneously injected once in STZ-diabetic mice prior to mating to restore euglycemia at early pregnancy. We mated STZ-diabetic female mice with the WT male mice 1 week after STZ injections.

### Quantitative Real-Time PCR

Total RNA was extracted from placental tissues using Trizol reagent (TransGen Biotech, Beijing, China) according to the manufacturer’s protocols. RNA quantity and purity were determined by a Nanodrop spectrophotometer (Thermo fisher, United States). For each sample, 1 μg of total RNA was reverse transcribed into cDNA using the TranScript All-in-One First-Strand SuperScript cDNA Synthesis Super Mix for qPCR (One-Step gDNA Removal) (TransGen Biotech, Beijing, China). Real-time PCR was performed using the AceQ Universal SYBR qPCR Master Mix (Vazyme Q511–02, Nanjing, China) on a QuantStudio 3 Real-Time PCR System (Thermo Fisher, United States). All primer sequences used in this analysis were provided in Supplemental Table S1.

Determination of the placental expression of FGF1, FGF4 and FGF21 in gestational diabetes patients, HFD induced or STZ induced GDM mice.

Human or mouse FGF1, FGF4 and FGF21 ELISA Kits (Cloud-Clone Corp, Houston, TX) were used to determine the expression levels of placental FGF1, FGF4 and FGF21 according to the manufacturer’s instructions. FGF standard and placental samples were reacted with assay buffer. Samples were detected at 405 nm absorbance for each sample by using ELISA plate.

### rFGF4 Treatment

A cDNA fragment encoding human FGF4 (Ala67-Leu206) was expressed and purified according to the published protocols (Bellosta et al., 2001; Ying et al., 2021). The pregnant GDM mice were divided into two groups and intraperitoneally injected with rFGF4 at 0.75 mg/kg at day E5.5, E7.5 and E9.5, E13.5 and E17.5 (E is the abbreviation of embryonic day) or 0.9% normal saline. All normal pregnant mice were treated with 0.9% normal saline at the same time as controls. The embryos and placentas were harvested for biochemical and molecular analyses on E10.5 or E17.5 after the mice was anaesthetized.

### Blood Glucose Measurement and Intraperitoneal Glucose Tolerance Test

Random blood glucose levels were determined at E0.5 and E10.5 using FreeStyle complete blood glucose monitor (Abbott Diabetes Care Inc., Alameda, CA, United States). For IPGTT, pregnant mice were fasted overnight at E8.5 and then injected intraperitoneally with glucose at a dose of 2 g/kg body weight. The blood glucose levels were measured prior to injection at 15, 30, 60, 90 and 120 min after glucose injection.

### Pathological, Histopathological and Immunofluorescent Evaluation of Mouse E10.5 Embryos

All E10.5 embryos were fixed in 4% paraformaldehyde overnight and embedded in paraffin. After deparaffinization and rehydration, paraffin sections (5 μm) were stained with haematoxylin and eosin (H&E) reagent using standard procedures. For immunofluorescence staining, paraffin sections were incubated with rabbit c-Caspase 3 antibody (Cell Signaling Technology; Cat#9664S; dilution: 1:1000) overnight at 4 °C, followed by incubation with secondary antibody (goat anti-rabbit IgG Alexa Fluor 488, Invitrogen, A11001, United States, 1:1000) for 1 h at room temperature. Thereafter, the sections were incubated with DAPI (4’,6-diamidino-2-phenylindole) staining (Southern Biotech, Birmingham, AL) for 10 min, and analyzed using a confocal microscope (Leica, Mannheim, Germany).

### Western Blot Analysis

Placental proteins were lysed with RIPA lysis buffer (25 mM Tris, pH 7.6, 150 mM NaCl, 1% NP40, 1% sodium deoxycholate, and 0.1% SDS) containing protease and phosphatase inhibitors (Thermo Fisher Scientific, United States). Protein concentrations were determined using a BCA Kit (Protein Assay Kit, Beyotime Biotechnology, Shanghai, China). The same amount of protein (60–100 μg) was separated electrophoretically using 12% SDS–PAGE and transferred to PVDF membranes (0.45 μm, Millipore, Germany). 10% non-fat milk in TBST was used to block the membrane at room temperature for 1 h. Each nitrocellulose membrane was incubated with antibodies to FGF4 (Abcam; Cat#ab106355 dilution: 1:1000), phospho-AKT (Cell Signaling Technology; Cat# 4060; dilution: 1:1000), AKT (Cell Signaling Technology; Cat#4691; dilution: 1:1000), P-AMPK-α (Cell Signaling Technology; Cat#2535; dilution: 1:1000), AMPK-α (Protein Tech; Cat#66536-1-lg; dilution: 1:1000), TNF-α (Cell Signaling Technology; Cat#3707; dilution: 1:1000), CD68 (Abcam; Cat#ab125212; dilution: 1:1000), GAPDH (Cell Signaling Technology; Cat#2118; dilution: 1:1000) at 4 °C overnight. After three washes with TBST, immune-reactive bands were detected by incubating with horseradish peroxidase (HRP) conjugated secondary antibodies (Santa Cruz Biotechnology; Cat#sc-2004 or Cat#sc-2005, dilution: 1:3000) at room temperature for 1 h. Protein bands were incubated using the EasySee Western Blot Kit (TransGen Biotech, Beijing, China) and visualized using an enhanced chemiluminescence (ECL) reagents (Bio-Rad, Hercules, CA, United States). Densitometric analysis was performed using ImageJ software (NIH, United States).

### TUNEL (Terminal Deoxynucleotidyl Transferase dUTP Nick End Labeling) Assay

Paraffin embryonic sections (5 μm) were performed using the One Step TUNEL Apoptosis Assay Kit (Green Fluorescence) (Beyontime C1086, Shanghai, China) according to the manufacturer’s protocols. Three embryos from three different mice per group were randomly selected and two sections per embryo were examined and visualized using a fluorescence microscope (TCS-SP8, Leica, Germany).

### Statistical Analysis

Data were presented as mean ± SEM. Each set of experiments were repeated independently at least three times with comparable results, and embryonic samples from each biological replicate were randomly taken from different mice. Student’s t-tests were performed to determine the significance of differences between pairs. One-way analysis of variance (ANOVA) was utilized to determine significant differences between multiple groups. Statistical significance was set at *p* < 0.05. The differences in NTD rates among experimental and control groups were examined with *χ2* Test.

## Results

### Patient Recruitment

A total of 37 women with normal glucose tolerance (n = 18) or GDM (n = 19) were included in this study. All women from the two groups were matched according to gestational age, height, body weight and BMI. The diastolic and systolic blood pressure across gestation in both normal and GDM pregnancies had no significant differences in the two groups. The fetal weights and the fetal biparietal diameter significantly increased in GDM patients compared with normal patients (*p* < 0.05) indicating gestational diabetes has effect to fetal growth. The glucose of OGTT at 0, 1 and 2 h were significantly higher than normal group (Table 1).

**TABLE 1:** Demographic characteristic of GDM and normal pregnant women.

Maternal			
Variables		Normal (*n* = 19)	GDM (*n* = 18)
Age (years)		26.4 ± 1.4 (24–29)	27.8 ± 3.4 (22–37)
Weight (kg)		65.5 ± 10.1 (49–90)	66.2 ± 4.4 (58.8–73.6)
Height (cm)		159.1 ± 3.7 (152–165)	157.9 ± 5.4 (149–173)
BMI (kg/m^2^)		25.9 ± 3.8 (19.6–35.2)	26.6 ± 2 (22.7–30.2)
OGTT at mid gestation (mmol/l)	0 h	4.3 ± 0.4 (3.5–4.9)	5.1 ± 0.7 (4.4–7.1) **
	1 h	6.1 ± 1.4 (4.5–8.8)	11 ± 1.7 (8.8–15.3) ****
	2 h	5.1 ± 0.6 (3.6–5.9)	10.2 ± 2 (8.4–16.6) ****
Gestational age at delivery (weeks)		39.2 ± 0.9 (37.6–40.9)	39.5 ± 1.0 (36.0–40.4)
Systolic Blood pressure (mm Hg)			
Mid gestation		117.1 ± 12.4 (90–135)	115 ± 10.6 (93–132)
Late gestation		115.8 ± 6.0 (105–127)	112.4 ± 8.4 (96–125)
Diastolic Blood pressure (mm Hg)			
Mid gestation		75.1 ± 7.8 (60–87)	74.9 ± 7.5 (60–87)
Late gestation		69 ± 6.6 (60–86)	70.7 ± 8.3 (51–89)
Newborn variables			
Placental weight (g)		511.1 ± 36.3 (450–560)	521.7 ± 74.7 (400–700)
Fetal weight (g)		3443.7 ± 334 (2940–4200)	3798.1 ± 455.2 (3050–4570)*
Biparietal diameter (mm)		89.9 ± 2.9 (85–94)	93.1 ± 2.7 (88–97) **
Head width (mm)		324.3 ± 9.3 (311–339)	329.4 ± 7.5 (311–338)
Fetal sex (male/female)		8/11	13/5

**p< 0.01, ****p< 0.0001, versus normal control.

### Endogenous Levels of FGFs and Their Physiological Correlations in Human GDM and Normal Gestational Placentas

**FIGURE 1:**
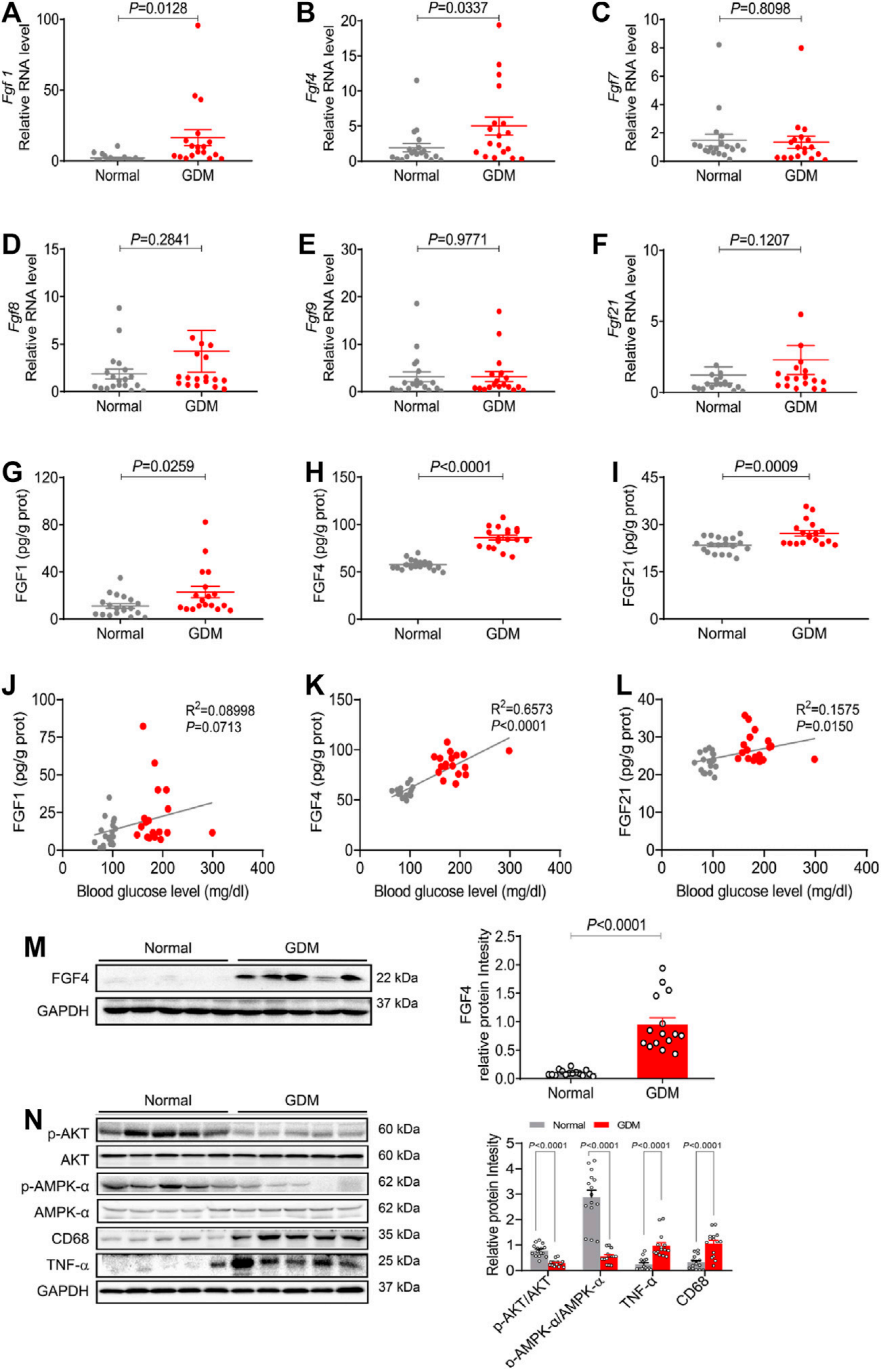
The endogenous expression of representative FGF family members and their physiological correlation in human GDM and normal gestational placentas **(A–F)** The mRNA levels of *Fgf1*, *Fgf4*, *Fgf7*, *Fgf8*, *Fgf9*, and *Fgf21* in placentas were analyzed using RT-PCR. **(G–I)** The FGF1, FGF4 and FGF21 expression levels were detected using ELISA analysis. **(J-L)** The correlation between growth factors (FGF1, FGF4, and FGF21) and blood glucose. Data are presented as mean values ±SEM (*n* = 18–19); **(M,N)** Protein levels of FGF4, phosphorylation of AKT (p-AKT), p-AMPK/AMPK, CD68 and TNF-α in human placentas were detected using western blotting. Data are presented as mean values ±SEM (*n* = 15); a value of *p* < 0.05 was considered to be statistically significant.

To determine the FGFs expression patterns in placentas of GDM during gestation, first, the placental RNA levels of six representative FGF members (*Fgf1*, *Fgf4*, *Fgf7*, *Fgf8*, *Fgf9* and *Fgf21*) were examined using 18 placentas of GDM patients and 19 placentas of normal patients. Compared to normal patients, mRNA expressions of *Fgf1* and *Fgf4* were significantly increased, but no differences of *Fgf7/8/9* and *Fgf21* expression levels in GDM patients (Figures 1.A–F). The protein levels of FGF1, FGF4 and FGF21 were notably increased in GDM patients, particularly FGF4 (Figures 1.G–I) by ELISA. Since endogenous FGF4 expression was most changed by PCR and ELISA in GDM patients, we also tested FGF4 expression at protein level by western blot and observed an increased expression of FGF4 in GDM patients, which is consistent with the results of RT-PCR and ELISA (Figure 1M) Interestingly, placental FGF4 levels were positively correlated (*R* ^*2*^ = 0.65; *p* < 0.0001) with blood glucose level in both GDM and normal patients, and the correlation is even higher than FGF21 (*R* ^*2*^ = 0.15; *p* < 0.01) (Figures 1J–L), suggesting that it may serve as a potential pathological marker of GDM.

We also analyzed the possible downstream mediators of signaling paths in placentas from GDM patients and normal patients. As the critical link to the insulin signaling pathway and inflammation signaling pathway, the expression levels of the phosphorylation of AKT (p-AKT) and inflammatory cytokines (TNF-α and CD68) were analyzed. Compared to normal patients, p-AKT expression was significantly decreased, whereas both CD68 and TNF-α were increased in placentas from GDM patients (Figures 1.N, P). Besides, AMPK (AMP-activated protein kinase) is necessary for the proper placental differentiation, nutrient transportation, maternal and fetal energy homeostasis, and the protection of fetal membrane during pregnancy. Nevertheless the maternal hyperglycemic environment might enrich cellular ATP to inactivate AMPK (Kumagai et al., 2018). Our data showed that phosphorylation of AMPK (p-AMPK) was significantly decreased in GDM placentas compared with normal gestational placentas (Figures 1.N, P). These results are consistent with previous findings that the insulin signaling pathway, inflammatory signaling pathway and the AMPK signal play important roles in the process of GDM (Barbour et al., 2007; Menon and Taylor, 2019; Carey et al., 2014; Kumagai et al., 2018).

### Placental Expression of Representative FGF Family Members in HFD Induced GDM Mice

**FIGURE 2:**
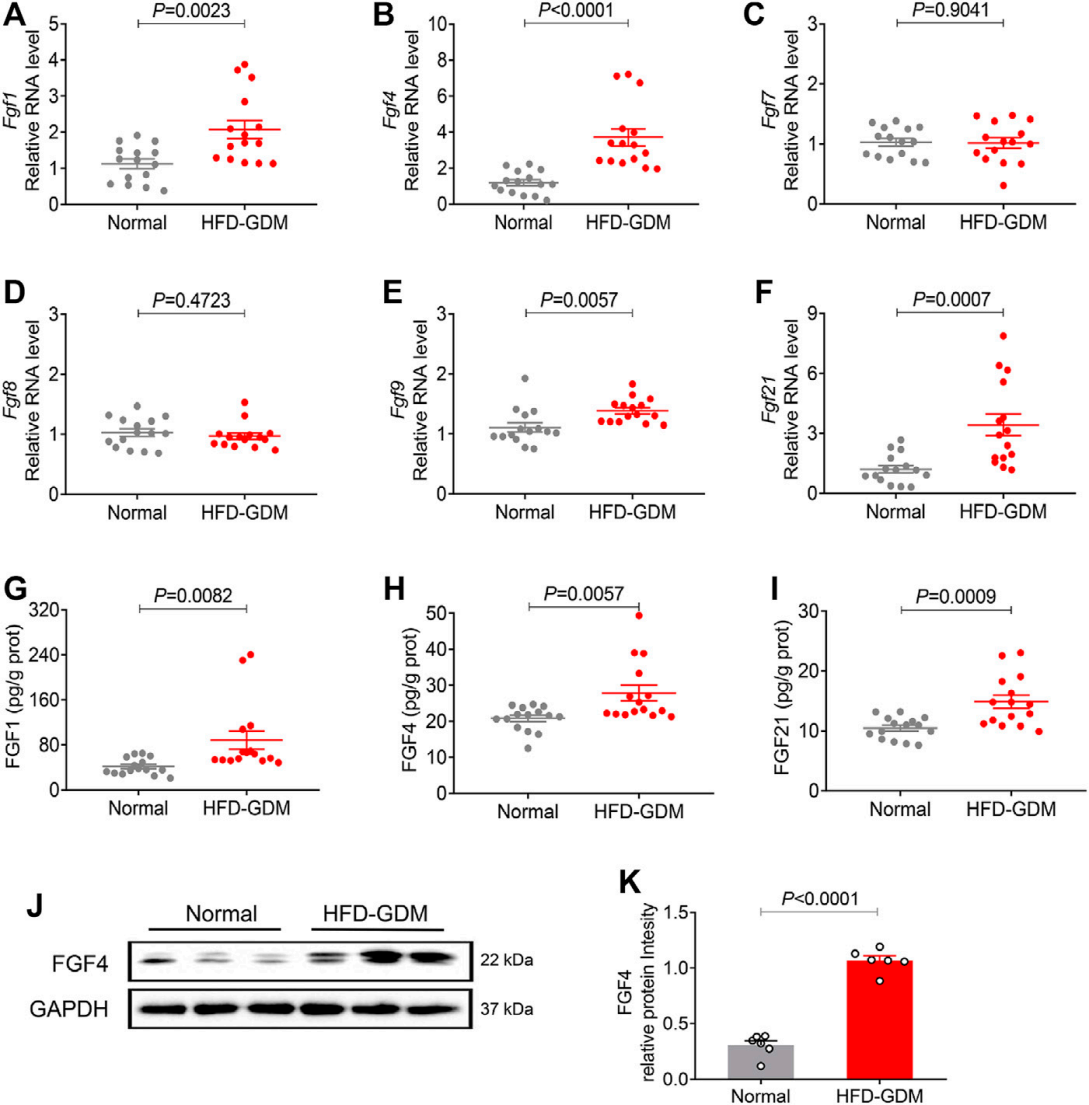
The mRNA and protein expression of representative FGF family members in placentas of HFD induced GDM animal models. **(A–F)** The mRNA levels of *Fgf1*, *Fgf4*, *Fgf7*, *Fgf8*, *Fgf9* and *Fgf21* were analyzed using RT-PCR. **(G–I)** The expression levels of placental FGF1, FGF4 and FGF21 were detected using ELISA. These experiments were performed using 15 placentas from different mice per group. Data are presented as mean values ±SEM (*n* = 15). **(J)** The FGF4 protein expression levels were detected using western blotting (three placenta from different mice per group), and representative images from two independent experiments were shown. **(K)** Densitometric quantification of western blots shown in **(J)**. Data are presented as mean values ±SEM (*n* = 6); a value of *p* < 0.05 was considered to be statistically significant.

We further explored the placental representative FGFs expression patterns from HFD induced gestational diabetes mice and normal c57bl/6 mice. Encouragingly, human-like FGF expression patterns in placentas were found from the HFD induced gestational diabetes mouse model (Figures 2.A–F). Compared to normal pregnant mice, the placental mRNA expressions of *Fgf4* and *Fgf21* were increased most in HFD-GDM mice, while no significant changes were found in the other FGFs like *Fgf7* and *Fgf8*. The protein levels of FGF1, FGF4 and FGF21 examined by ELISA also revealed a significant increase in HFD-GDM mice especially FGF4 (Figures 2.G–I). Moreover, western blot analysis also showed an increased FGF4 expression in HFD-GDM mice which is consistent with the findings in RT-PCR and ELISA (Figures 2.J–K).

### Placental Expression of Representative FGF Family Members in STZ Induced GDM Mice

**FIGURE 3:**
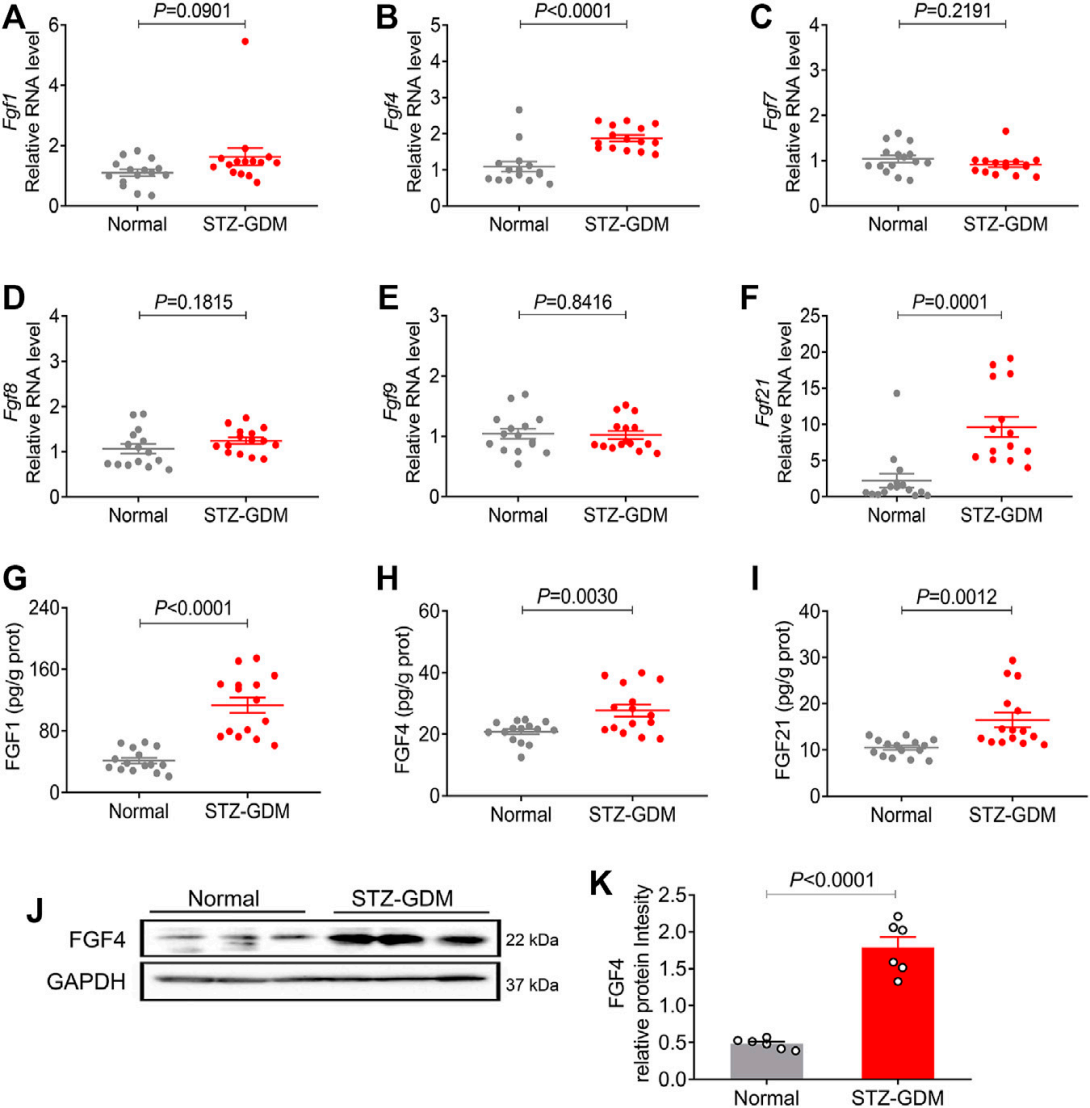
The mRNA and protein expression of representative FGF family members in placentas of STZ induced GDM animal models. (**A–F**) The mRNA levels of *Fgf1*, *Fgf4*, *Fgf7*, *Fgf8*, *Fgf9*, and *Fgf21* were analyzed using RT-PCR. **(G–I)** The protein expression levels of the placental FGF1, FGF4 and FGF21 were detected using ELISA kit. These experiments were performed using fifteen placentas from different mice per group. Data are presented as mean values ±SEM (*n* = 15). **(J)** The FGF4 protein expression levels were detected using western blotting (three placentas from different mice per group), and representative images from two independent experiments were shown. **(K)** Densitometric quantification of western blots shown in **(J)**. Data are presented as mean values ±SEM (*n* = 6); a value of *p* < 0.05 was considered to be statistically significant.

Furthermore, we established another GDM mouse model induced by STZ as previously reported to see which model better mimic the human gestational diabetes. STZ-GDM mice also showed similar patterns of placental FGFs expression profiles (Figures 3A–F). The mRNA and protein expression of three FGFs (FGF1, FGF4 and FGF21) were increased in STZ-GDM mice compared to normal group, whereas no significant changes in FGF7, FGF8 and FGF9 (Figures 3G–I). Western blotting analysis also revealed the increased protein expression level of FGF4 (Figures 3J–K). Taken a whole, the human and animal studies consistently demonstrate that there is a correlation between paracrine FGF4 and GDM. FGF4 may play some role in the etiology of GDM.

### rFGF4 Treatment Normalizes the Adverse Metabolic Phenotypes and Partially Alleviates Embryonic NTD Formation in HFD Induced GDM Mice

**FIGURE 4:**
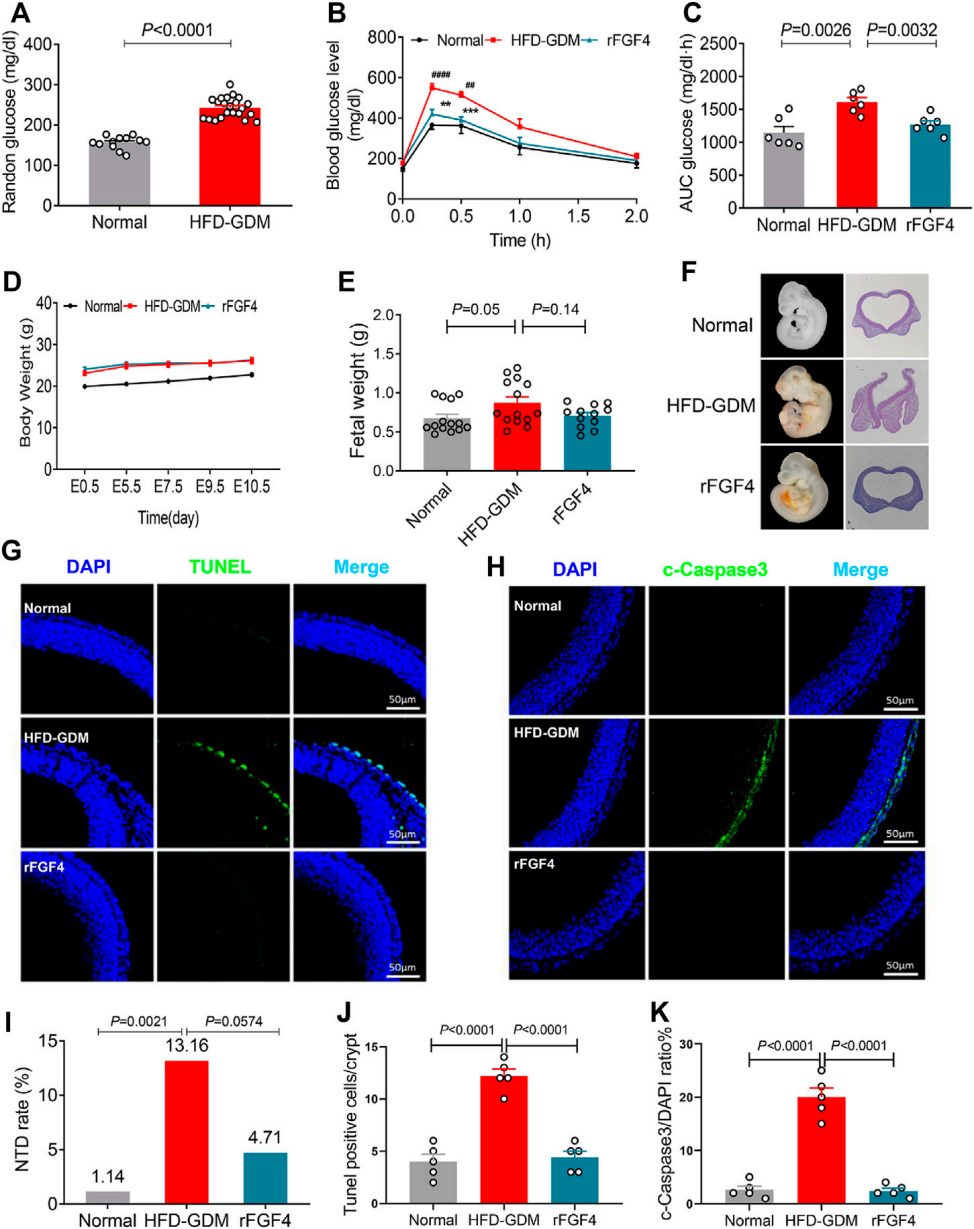
rFGF4 treatment normalizes the adverse metabolic phenotypes and alleviates maternal HFD–induced NTD formation. **(A)** Random blood glucose levels of the normal and HFD-GDM mice (*n* = 10–11). **(B–C)** A glucose tolerance test (GTT) done **(B)** and the accompanying integrated area under curve (AUC) for changes in blood glucose **(C)** at E9.5 (*n* = 6). ^##^ *p* < 0.01, ^####^ *p* < 0.0001 versus normal mice control; **p < 0.01, ***p < 0.001 versus HDF-GDM mice. **(D)** Body weight changes of each group mice. **(E)** Embryos weight at E17.5 (*n* = 4). **(F)** Representative closed and open neural tube structures of E10.5 embryos from control, HFD induced GDM and rFGF4-treated mice. **(G,J)** Representative images of the TUNEL assay showing apoptotic cells (green signal) and quantification of TUNEL positive cells in **(G)**. Cell nuclei were stained with DAPI (blue). Scale bar = 50 μm. (*n* = 5). **(I)** NTD formation rates of embryos from control, HFD induced GDM and rFGF4-treated mice. **(H,K)** Representative immunofluorescence staining and quantification of cleaved-Caspase 3 in E10.5 embryos from control, HFD induced GDM and rFGF4-treated mice. Scale bar = 50 μm (*n* = 5).

To further confirm the role of FGF4 in GDM, recombinant FGF4 were engineered, expressed and purified to examine whether FGF4 replenishment could regulate the adverse metabolic phenotypes of HFD induce gestational diabetic mice and protect fetus or not, GTT (glucose tolerant test) was performed since glucose intolerance is one of the well-defined characteristics of GDM. Notably, HFD induced GDM mice have the elevated random glucose levels (Figure 4.A). The blood glucose levels of HFD-GDM mice were significantly reduced after rFGF4 treatment at 15 and 30 min during GTT (Figure 4B). the accompanying integrated area under curve (AUC) for changes in blood glucose in rFGF4-treated GDM mice was close to the level observed in normal diet-fed mice (Figure 4C). Therefore, our data suggest that rFGF4 treatment ameliorates diabetic phenotypes in HFD-GDM mice without causing any significant change in body weight (Figure 4D).

To determine whether rFGF4 can protect the fetus, we examined the embryo weight and embryonic NTDs in the presence and absence of rFGF4 in HFD-GDM mice. From analysis of the embryo weight at day 17.5, we could see that rFGF4 treatment somewhat slowed down the embryo over growth but it had no significantly difference between rFGF4 treatment and no treatment (*p* = 0.14) (Figure 4E). The embryonic NTD rate from the rFGF4-treated GDM mice was 4.71%, which was significantly lower than that in embryos from the non-rFGF4-treated GDM mice (13.16%). In addition, our data suggest that rFGF4 reduced the resorptions in GDM mice but it might not completely prevent maternal diabetes-induced resorptions (Figures 4F, I **,** Table 2; Supplemental Figure S2). Moreover, rFGF4 treatment alleviated neuroepithelial cell apoptosis by reducing levels of cleavage of Caspase-3 (Figures 4G-H, J, K).

**TABLE 2:** rFGF4 treatment alleviates NTD formation of HFD induced GDM mice.

Group	Total number of embryos	Resorption rate (%)	NTD rate (%)
Normal (*n* = 11)	90	2.22 (2)	1.14 (1)
HFD-GDM (*n* = 10)	84	9.52 (8) #	13.16 (10) ##
HFD-GDM + rFGF4 (*n* = 11)	91	6.59 (6)	4.71 (4)

Normal group fed regular diet; HFD-GDM, group fed 60% fat diet; HFD-GDM + rFGF4 group fed HFD, and treated with rFGF4 during pregnancy. In brackets are the numbers of resorted or NTD, embryos. Statistical differences were analyzed by the χ2 test. ^#^ *p* < 0.05, ^##^ *p* < 0.01 versus normal mice control.

### rFGF4 Alleviates Embryonic NTD Formation and Resorption, but has No Anti-hyperglycemic Effect in STZ Induced Gestational Diabetes Mice

**FIGURE 5:**
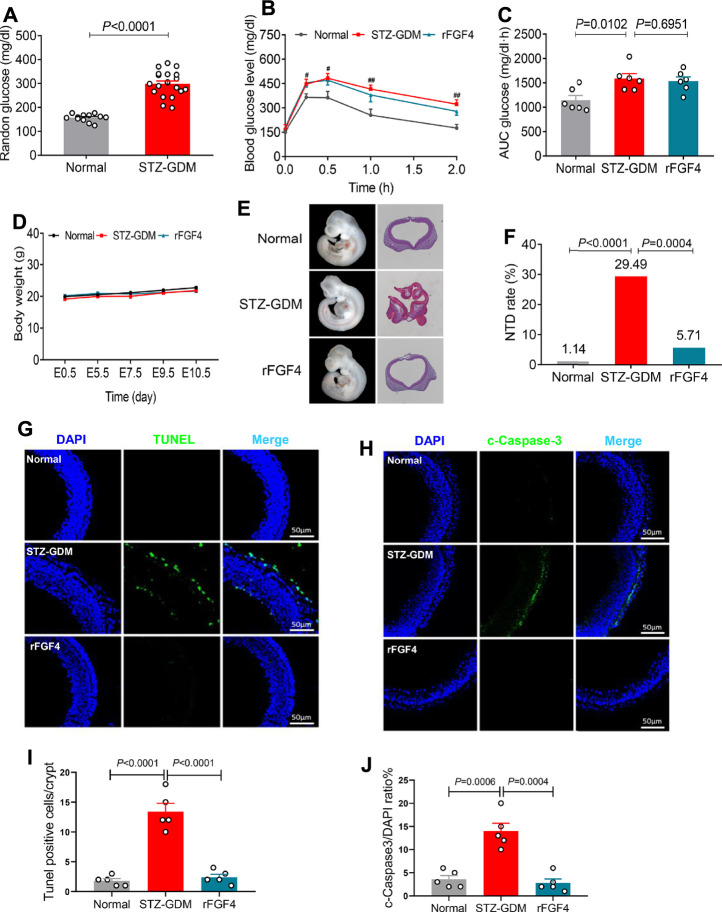
rFGF4 treatment has no effect of glucose lowering in STZ induced mice, but it alleviates STZ induced NTD formation and resorption. **(A)** Random blood glucose levels in normal and STZ induced GDM groups (*n* = 11). **(B–C)** A glucose tolerance test (GTT) done **(B)** and the accompanying integrated area under curve (AUC) for changes in blood glucose **(C)** at E9.5 (*n* = 6). ^#^ *p* < 0.05, ^##^ *p* < 0.01 STZ-GDM versus normal mice. **(D)** Body weight changes of each group mice. **(E)** Closed and open neural tube structures of E10.5 embryos from control, STZ induced GDM and rFGF4-treated mice. **(F)** NTD formation rates of embryos from control, STZ induced GDM and rFGF4-treated groups. **(G)** TUNEL assay showing apoptotic cells (green signal). Cell nuclei were stained with DAPI (blue). Scale bar = 50 μm. **(I)** Quantification of TUNEL positive cells in **(G)** (*n* = 5). **(H,J)** Immunofluorescence staining and quantification of cleaved-Caspase 3 in E10.5 embryos from control, STZ induced GDM and rFGF4-treated mice. Scale bar = 50 μm (n = 5).

We also investigated the effects of rFGF4 on adverse glucose regulation and embryo protection in STZ induced gestational diabetes mice. rFGF4 treatment cannot improve glucose tolerance in STZ induced GDM mice (Figures 5A–C). There was also no significant change in body weight (Figure 5.D) or embryo weight at day E17.5 (data not shown). However, our data showed that the resorption and NTD rates were reduced in the rFGF4-treated GDM mice (Figure 5F, Table 3; Supplemental Figure S2). TUNEL and immunostaining analysis showed that rFGF4 treatment alleviated neuro-epithelial cell apoptosis by reducing the levels of cleavage of Caspase-3 (Figures 5G–J).

**TABLE 3:** FGF4 treatment alleviates maternal STZ induced GDM–induced NTD formation.

Group	Number of embryos	Resorption rate (%)	NTD rate (%)
Ctrl (*n*=12)	101	5.94 (6)	2.97 (3)
T1D (*n*=11)	83	8.43 (7)*	24.10 (20)*
T1D+331 (*n*=9)	73	6.85 (5)	10.96 (8)

Data are mean ± SEM unless otherwise indicated. Ctrl group fed normal diet, T1D group were given STZ before pregnancy. *Significant differences compared with the other two groups as analyzed by the turkey test or T test.

### rFGF4 Suppressed Inflammation Signaling Pathways of Placenta in Both Two Gestational Diabetes Mice Model

**FIGURE 6:**
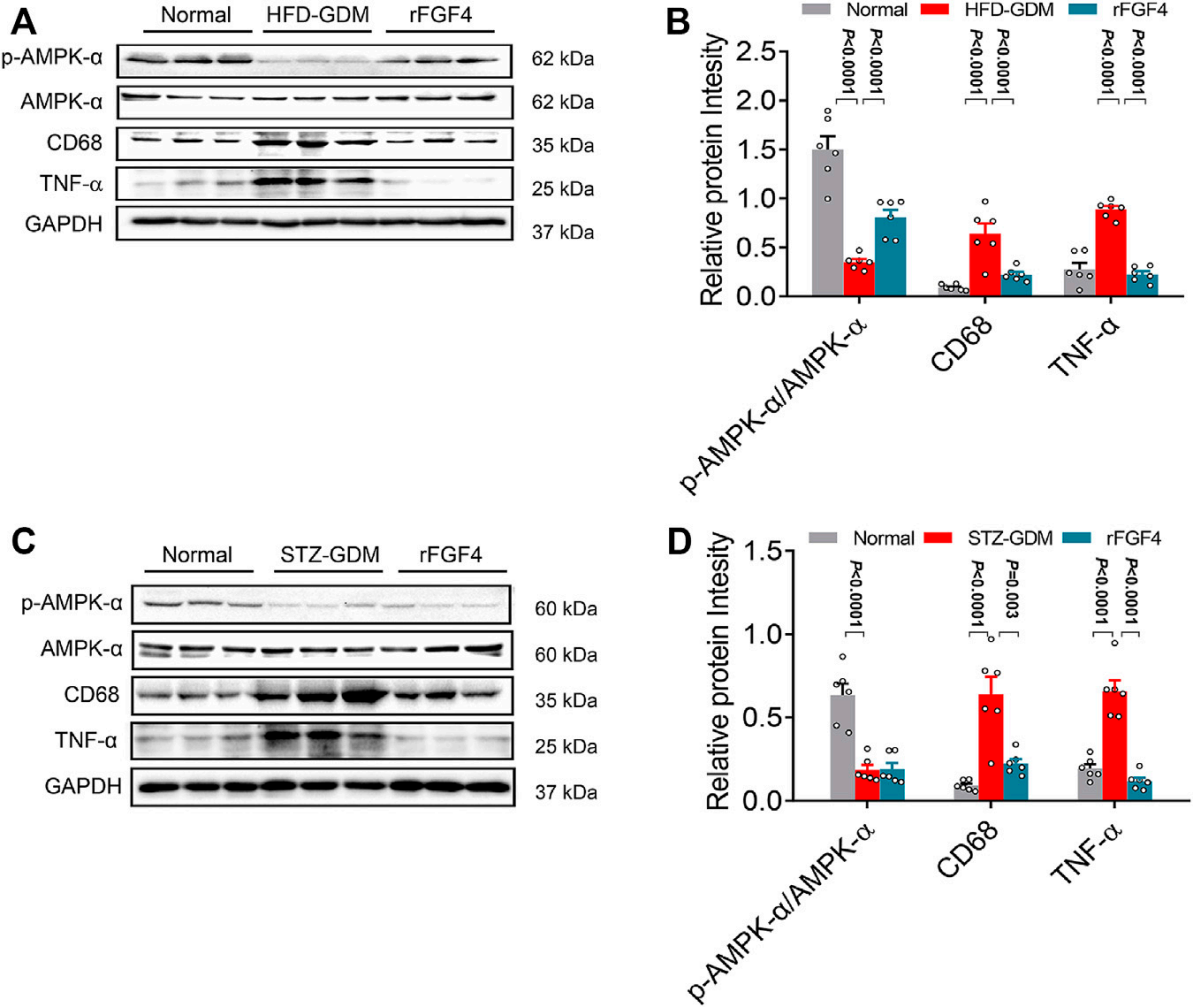
Possible mechanism of rFGF4 treatment in two different GDM mice model. **(A)** Protein levels of phosphorylation of AKT (p-AKT), p-AMPK/AMPK, CD68 and TNF-α in the placentas of control, HFD induced GDM and rFGF4-treated mice. **(B)** Densitometric quantification of western blots shown in **(A)**. Data are presented as mean values ±SEM (*n* = 6); a value of *p* < 0.05 was considered to be statistically significant. **(C)** Protein levels of phosphorylation of p-AMPK/AMPK, CD68 and TNF-α in the placentas of control, STZ induced GDM and rFGF4-treated mice. **(D)** Densitometric quantification of western blots shown in **(C)**. Data are presented as mean values ±SEM (*n* = 6); a value of *p* < 0.05 was considered to be statistically significant.

Then the possible downstream mediators of insulin signaling pathway, inflammatory signaling pathway and AMPK phosphorylation related to the pathogenesis of GDM were analyzed. Remarkably, rFGF4 treatment inhibited inflammation cytokines TNF-α and CD68 and effectively activated p-AMPK, but could not active AKT in HFD induced gestational diabetes mice (Figures 6A, B and Supplemental Figure S1), indicating that it may have no effect on insulin pathway signaling. However, in placentas of STZ induced gestational diabetes mice, rFGF4 treatment had no effect on the AMPK and AKT signaling. But it still inhibited the placental inflammatory cytokines TNF-α and CD68. (Figures 6C, D and Supplemental Figure S1). Taken together, our data support the role of rFGF4 in placentas likely via suppressing inflammation signaling pathways, and independent of glucose lowering.

## Discussion

Here, we performed a systematic screening of representative growth factors from six different FGF subfamilies in order to investigate their expression changes in GDM. Strikingly, we first time demonstrated that paracrine FGF4 was significantly expressed in GDM patients. Remarkably, we also found a significant positive correlation between FGF4 and blood hyperglycemia, suggesting that it can be a potent and potentially indicator for GDM (Figure 1). Our two GDM mice model studies consistently confirmed that FGF4 is highly effective in lowering NTDs, inhibiting proinflammatory signaling cascades and apoptotic pathway in mouse placentas (Figures 3, 5).

Since the introduction of alpha-fetoprotein screening in the late 1970s to identify women at increased risk of having a fetus with a neural tube defect (NTD), an association has been found accompany with pregnancy complications. In our study, we found that rFGF4 is highly effective in lowering NTDs, and the expression of endogenous FGF4 is up-regulated in GDM. So whether FGF4 can become a potential marker of NTDs and its mechanism deserve further study.

While the specific etiology and pathogenesis of GDM are not fully understood, the disease is thought to arise as a result of insulin resistance, inflammation, placental hormones, and genetic factors (Tatjana and Ali, 2003; Barbour et al., 2007; Kumagai et al., 2018; Menon and Taylor, 2019). Previous studies have shown that FGF21 is an important metabolic regulator in the progression of GDM (Kharitonenkov et al., 2005; Coate et al., 2017). Stein group was first to report the circulating FGF21 is significantly higher in patients in the second and third tertile of HOMA-IR as compared with the first tertile. But in that study the patients with GDM were significantly older as compared with control subjects (Stein et al., 2010). Further studies also showed that the serum and the placental FGF21 levels were increased in GDM patients although using different criteria of the involvement of GDM patients (Dekker Nitert et al., 2014b; Li et al., 2015; Megia et al., 2015). Here, we also determined the placental FGF21 expression levels, and found that the FGF21 expression level was increased in pregnant women with GDM. Moreover, we have also found that the FGF21 level is positively correlated with blood glucose levels (Figures 1F,L).

FGF family is divided into endocrine FGFs and paracrine FGFs, and endocrine type FGF19/21 has been widely studied in the field of metabolism. Mounting evidence showed that FGFs interact with FGFR in the 1:1 FGF-FGFR complex to facilitate FGF-FGFR dimerization, with increased intracellular receptor tyrosine kinase activity that triggers various downstream signaling pathways (Huang et al., 2017; Zinkle and Mohammadi, 2018; Niu et al., 2020). So these endocrine/paracrine FGFs may also share the same mode of action and regulated the development of GDM. However, paracrine FGFs were reported to be associated with regeneration, development and angiogenesis, but not related to metabolism. So that is why FGF4 (a paracrine FGF) has not been observed as GDM dependent variable growth factor at early time. In recent years, paracrine FGFs have been found to play an important role in metabolic regulation. Therefore, we selected representative members of six FGFs family members for systematic analysis, and for first time discovered that paracrine FGF4 were greatly up-regulated in GDM, indicating its crucial role in the progression of GDM.

Paracrine FGF4 is an indispensable player in various stages of embryonic development, including implantation, morphogenesis, and organogenesis (Boulet and Capecchi, 2012; Moon et al., 2000; Ryan et al., 2019; Yasui et al., 2014; Fukuyama et al., 2007). Our animal studies further provide clear evidence that FGF4 has a certain protective effect on the fetus in GDM condition. AMPK maintains the maternal metabolic balance and protects fetal growth from diverse types of stress throughout the pregnancy (Kumagai et al., 2018). Consistent with the previous literature (Martino et al., 2015), we found that the AMPK gene expression was suppressed in the placentas of GDM women. Meanwhile rFGF4 treatment activated p-AMPK in HFD induced GDM mice, but not in the STZ induced GDM mice, probably because of the two mouse models might have different pathogenesis, or the rFGF4 protection was independent of AMPK activation. In addition, GDM could elicit major changes in the transcriptomic profiles in placentas particularly the prominent increase of inflammatory markers and mediators (Tatjana and Ali, 2003). Interestingly, our data provides the first evidence that rFGF4 treatment reduced placental inflammation in both animal models of GDM supported by the significant reduction of inflammatory markers such as TNF-α (Figure 6). Notably, TNF-α has been previously recognized as the most prominent factor contributing to insulin resistance in both diabetes and diabetes pregnancy (Hotamisligil et al., 1993; Uysal et al., 1997; Kirwan et al., 2002).

Collectively, our study provides new insights into the important roles of paracrine FGF4 in the progression of GDM. FGF4 may be used as a promising pathological marker of GDM. And rFGF4 exerts an intervention on GDM in a manner that is independent of glucose control and directly inhibits inflammation with consequent reduction of neuroepithelial cell apoptosis and NTD. It is worth mentioning that in future to perform a systematic analysis on the detailed biological roles of the FGF family members at different time points throughout the development of GDM would be interesting.

## Data Availability

The original contributions presented in the study are included in the article/Supplementary Material, further inquiries can be directed to the corresponding authors.
