# Supplementary material for: FGF4, A New Potential Regulator in Gestational Diabetes Mellitus
Source: Front Pharmacol. 2022 Mar 4;13:827617. doi: 10.3389/fphar.2022.827617 (PMC8934430; doi:10.3389/fphar.2022.827617)
Supplement: Supplementary file 1 [file DataSheet1.docx]

**SUPPLEMETAL INFORMATION**

**FGF4, a new potential regulator in gestational diabetes mellitus**

**
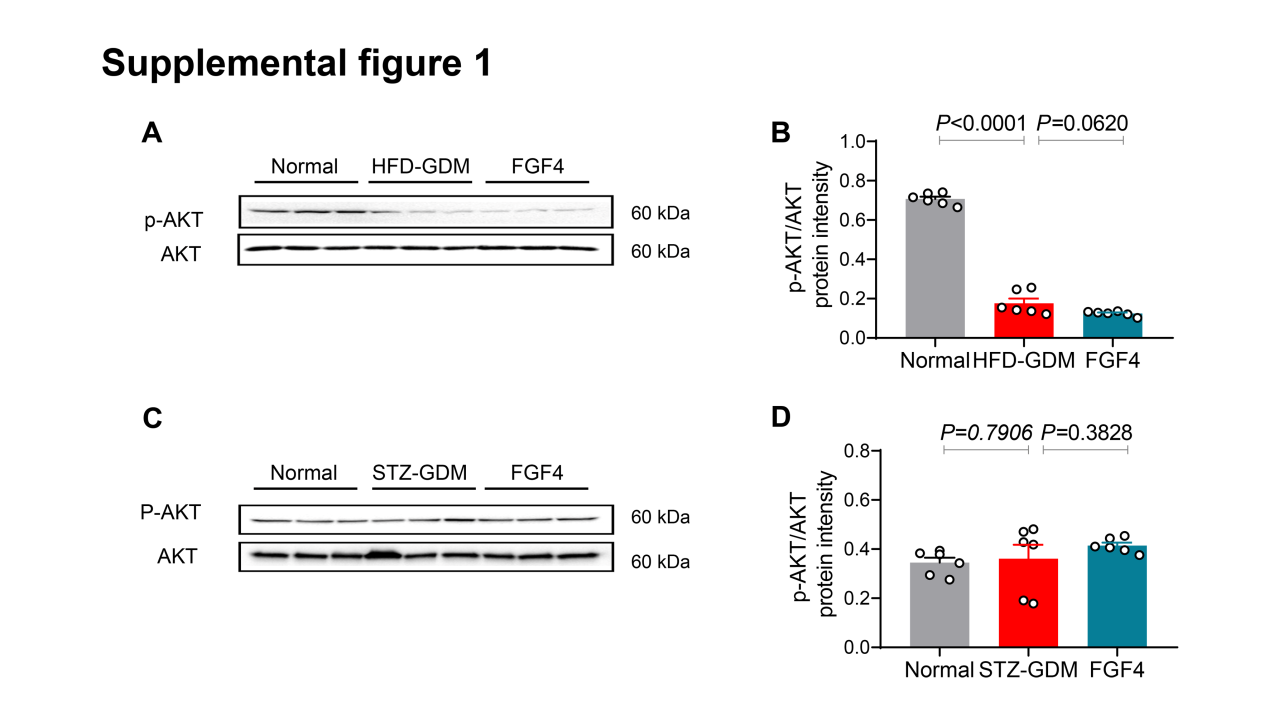
**

**Supplemental Figure 1. rFGF4 treatment could not active p-AKT in HFD induced GDM and STZ induced GDM placenta.**

(**A**) The AKT phosphorylation level in the placentas of control, HFD-induced GDM and rFGF4-treated mice. (**B**) Densitometric quantiﬁcation results of (**A**) using image J software. (**C**) The AKT phosphorylation level in the placentas of control, STZ-induced GDM and rFGF4-treated STZ-GDM mice. (**D**) Densitometric quantiﬁcation results of (**C**) using image J software. Data were presented as mean ± SEM (n=6). A value of P<0.05 was considered to be statistically significant.

**
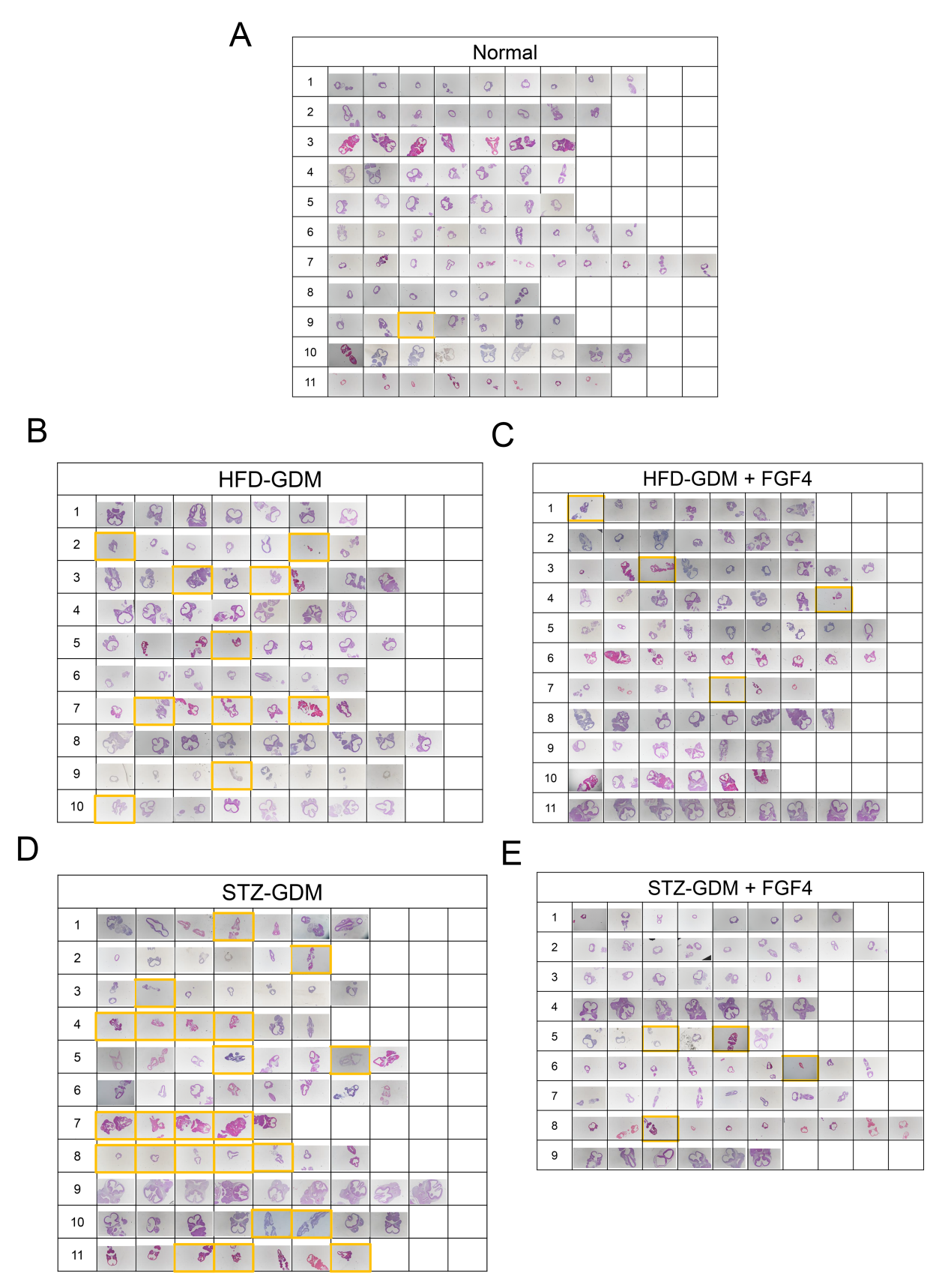
**

**Supplemental Figure 2. Hematoxylin and eosin (H&E)-stained images of the closed and open neural tubes of all E10.5 embryos from different groups.**

(**A**) The neural tube images of all E10.5 embryos from normal mice (n=90). (**B**) The neural tube images of all E10.5 embryos from whole HDF-GDM mice (n=84). (**C**) The neural tube images of all E10.5 embryos from HDF-GDM mice FGF4 treated (n=91). (**D**) The neural tube images of all E10.5 embryos from STZ-GDM mice (n=86). (**E**) The neural tube images of all E10.5 embryos from STZ-GDM mice treated FGF4 (n=74). The numbers in the left column represent the number of individual mice, whereas the horizontal rows represent the neural tube images of E10.5 embryos for each mouse. The neural tube malformations were labeled in yellow.

**Supplemental Table 1. Primer information**

| Transcript | Primers | Origin |
| --- | --- | --- |
| GAPDH | F: AGGTCGGTGTGAACGGATTTG | human |
|  | R: GGGGTCGTTGATGGCAACA |  |
| *Fgf1* | F:GAAGCCCAAACTCCTCTACTGTAGC | human |
|  | R: TGTTGTAATGGTTGTGGTCCAGC |  |
| *Fgf4* | F: CTCGCCCTTCTTCACCGATG | human |
|  | R: GTAGGACTCGTAGGCGTTGTA |  |
| *Fgf7* | F: TCCTGCCAACTTTGCTCTACA | human |
|  | R: CAGGGCTGGAACAGTTCACAT |  |
| *Fgf8* | F: CGAGACCGACACCTTTGG | human |
|  | R: TCCTTGCCTTTGCCGTTAC |  |
| *Fgf9* | F: CAGGCGGAGGCAGCTATAC | human |
|  | R:CCTGGTTCCCTGGATAGTACC |  |
| *Fgf21* | F: ATGGATCGCTCCACTTTGACC | human |
|  | R: GGGCTTCGGACTGGTAAACAT |  |
| GAPDH | F: TGACCTCAACTACATGGTCTACA | mouse |
|  | R: CTTCCCATTCTCGGCCTTG |  |
| *Fgf1* | F: CCCTGACCGAGAGGTTCAAC | mouse |
|  | R: GTCCCTTGTCCCATCCACG |  |
| *Fgf4* | F: TGGGCCTCAAAAGGCTTCG | mouse |
|  | R: CGTCGGTAAAGAAAGGCACAC |  |
| *Fgf7* | F: ACCTGAGGATTGACAAACGAGG | mouse |
|  | R: CCACGGTCCTGATTTCCATGA |  |
| *Fgf8* | F: CCGAGGAGGGATCTAAGGAAC | mouse |
|  | R: CTTCCAAAAGTATCGGTCTCCAC |  |
| *Fgf9* | F: CCCAACGGTACTATCCAGGGA | mouse |
|  | R: AGGCCCACTGCTATACTGATAAA |  |
| *Fgf21* | F: CTGGGGGTCTACCAAGCATA | mouse |
|  | R: CACCCAGGATTTGAATGACC |  |
